# Paracrine senescence of human endometrial mesenchymal stem cells: a role for the insulin-like growth factor binding protein 3

**DOI:** 10.18632/aging.102737

**Published:** 2020-01-17

**Authors:** Irina Vassilieva, Vera Kosheverova, Mikhail Vitte, Rimma Kamentseva, Alla Shatrova, Natalia Tsupkina, Elena Skvortsova, Aleksandra Borodkina, Elena Tolkunova, Nikolay Nikolsky, Elena Burova

**Affiliations:** 1 Department of Intracellular Signaling and Transport, Institute of Cytology of the Russian Academy of Sciences, St. Petersburg 194064, Russia

**Keywords:** endometrial stem cells, paracrine senescence, secretome, IGFBP3, endocytosis

## Abstract

Stress-induced premature cell senescence is well recognized to be accompanied by emerging the senescence-associated secretory phenotype (SASP). Secreted SASP factors can promote the senescence of normal neighboring cells through autocrine/paracrine pathways and regulate the senescence response, as well. Regarding human endometrium-derived mesenchymal stem cells (MESCs), the SASP regulation mechanisms as well as paracrine activity of senescent cells have not been studied yet. Here, we examined the role of insulin-like growth factor binding protein 3 (IGFBP3) in the paracrine senescence induction in young MESCs. The H_2_O_2_-induced premature senescence of MESCs led to increased IGFBP3 in conditioned media (CM). The inhibitory analysis of both MAPK and PI3K signaling pathways showed that IGFBP3 releasing from senescent cells is mainly regulated by PI3K/Akt pathway activity. IGFBP3 appears to be an important senescence-mediating factor as its immunodepletion from the senescent CM weakened the pro-senescent effect of CM on young MESCs and promoted their growth. In contrast, young MESCs acquired the senescence phenotype in response to simultaneous addition of recombinant IGFBP3 (rIGFBP3). The mechanism of extracellular IGFBP3 internalization was also revealed. The present study is the first to demonstrate a significant role of extracellular IGFBP3 in paracrine senescence induction of young MESCs.

## INTRODUCTION

The insulin-like growth factor (IGF) binding proteins (IGFBPs) belong to a superfamily of six high-affinity IGF-binding proteins [1–3]. The IGFBP family members display high sequence homology however each of the IGFBPs has unique structural features [1–3]. Being secreted into the extracellular environment, the IGFBPs inhibit or stimulate cell growth by altering an accessibility of the extracellular IGFs to IGF receptors on the cell surface [3–5]. The IGFs are bound with IGFBPs either in a binary complex or a ternary complex contained IGF, IGFBP3 (rarely IGFBP5), and a glycoprotein called acid labile subunit (ALS) [1]. In addition to their ability to suppress or enhance IGF actions, the IGFBPs are capable of influencing cell proliferation, migration, differentiation, angiogenesis, and apoptosis in IGF/IGF-1R independent manner [2, 5].

IGFBP3 is a secreted glycoprotein that has multiple roles both outside and inside the cell. In the serum, it circulates as a 150-kDa ternary complex with IGF mitogenic peptides, principally IGF-I, and ALS, protecting them from rapid degradation, and regulates their bioavailability [1, 3]. Despite of IGFBP3 is known to regulate cell growth by reversibly sequestering extracellular IGF-I [1, 3], accumulating evidence demonstrates also IGF-independent effects of IGFBP3 via interaction with various binding proteins on the cell surface and within cells [1, 4, 5]. In cancer cells, IGFBP3 internalization was shown to be mediated by either caveolae or clathrin endocytic pathway via binding to transferrin/transferrin receptor complexes [4]. Also, the type V TGF-β receptor and the low-density lipoprotein receptor-related protein-1/α_2_M receptor have been proposed as the cognate cell surface receptors for IGFBP3 [5, 6].

Although IGFBP3 is a secreted protein, it contains a nuclear localization signal (NLS) and is detected in the nucleus of multiple cell lines [6, 8, 9]. The IGFBP3 nuclear import is mediated by binding to importin-β which interacts with NLS domain in the IGFBP3 C-terminus [7, 8]. Despite the evidence presented, it still remains unresolved whether the IGFBP3 secretion is required for its nuclear localization [6, 8, 9]. Nuclear IGFBP3 by interacting with the retinoid X receptor and other nuclear hormone receptors can regulate transcriptional activity [10–12]. Nuclear transport of IGFBP3 may be required for its pro-apoptotic activity [6, 8, 10, 13] however the alternative pathways of IGFBP3-induced apoptosis, independent of its nuclear entry, also exist [9, 14, 15]. Apart from well-documented pro-apoptotic and growth-inhibitory functions of IGFBP3, evidence is emerging that it exhibits also pro-survival and growth-promoting properties in a variety of cancers [16–20]*.* According to Baxter, IGFBP3, acting at the crossroads between cell death and cell survival, can serve as a “caretaker”, contributing to the repair of damaged DNA, as well as a “gatekeeper”, preventing cell replication and promoting cell death when genomic integrity is compromised [17].

Currently, there is increasing evidence that the IGFBPs have an important role in controlling cell senescence independent of IGFs [21–26]. Senescent cells release senescence-associated secretory phenotype (SASP) proteins to execute several functions such as sensitizing neighboring cells to senescence, immunomodulation, promoting tissue repair, and impairing or fostering cancer growth. Progress in understanding the mechanisms of the SASP regulation has been reviewed [27–31]. The secretome composition comprises a broad repertoire of SASP factors, including growth regulators, pro-inflammatory cytokines such as interleukins and chemokines, proteases, extracellular matrix proteins etc., and depends on both genotoxic stress and cell type. Recent studies have provided evidence that SASP factors via autocrine/paracrine pathways may affect neighboring cells inducing their senescence [22, 30, 32–36].

Mesenchymal stem cells (MSC) are multipotent cells with a substantial potential in human regenerative medicine due to their ability to migrate to sites of injury and capability to suppress immune response. While it was initially hypothesized that replacement of damaged cells is an important mechanism of transplanted MSC action, focus has shifted to their paracrine actions due to secreted factors that support regenerative processes in the damaged tissue, induce angiogenesis and modulate immune system. Thus, the paracrine activity of MSC is supposed to underlie the efficiency of MSC-based therapy. To date, many impressive results regarding the use of MSC-based therapy for treatment cardiovascular and rheumatic diseases, bone disorders, neuronal injury, diabetes, etc. are obtained [37–41]. Senescence causes profound alterations in the secretome composition [22, 24, 32] and therefore impairs one of the key MSC biological functions [42, 43]. In this regard, the SASP-dependent regulation mechanism of cellular senescence is a current topic of MSC biology research.

Human endometrium-derived mesenchymal stem cells (MESCs) are an easily available source of adult stem cells [44, 45]. Their differentiation abilities, high proliferation activity during long-term cultivation, genetic stability, lack of tumorigenicity, and low immunogenicity make MESCs promising cell therapy candidates. Currently, cultured MESCs are applied in clinical trials, and encouraging results have been reported [46, 47]. To improve the efficiency of MESCs transplantation, it should be considered a possibility of their premature senescence under oxidative stress [48], arising commonly at lesion areas. In this case, the SASP factors of senescent MESCs can induce the premature senescence program in surrounding cells that results in a loss of their ability to regenerate damaged tissues.

Recently, we have shown that SASP factors secreted by senescent MESCs to conditioned medium (CM) are capable to trigger premature senescence in young cells [49]. The molecular mechanisms of SASP regulation as well as a paracrine activity of senescent cells towards senescence propagation in MESCs culture have not been studied yet. By applying the proteomic analysis of senescent MESCs secretome, up-regulation of IGFBP3 involved in SASP was found (data publishing in progress). In this regard, the present study is aimed to reveal a potential role for IGFBP3 in paracrine senescence induction within the MESCs culture. To the best of our knowledge, the senescence-inducing action of IGFBP3 towards MESCs remains still unexplored. Also, we have analyzed a functional status of pathways regulating both IGFBP3 secretion by senescent cells and its entry the young cells.

## RESULTS

In previous studies, we have demonstrated that MESCs undergo a premature senescence in response to sublethal H_2_O_2_ doses [50, 51] while secreting the SASP factors to conditioned media (CM). It was also shown that CM acquires the senescence-inducing properties due to accumulation of secreted factors during senescence, and may trigger senescence in young MESCs [49]. According to our data obtained with applying high-resolution mass spectrometry, among SASP factors secreted by MESCs the upregulated IGFBP3 and PAI-1 have been identified. In the current work, we have investigated phenomenon of IGFBP3 secretion by senescent cells and an impact of extracellular IGFBP3 on paracrine senescence induction in young cells, as well.

### Increased extracellular IGFBP3 in response to H_2_O_2_-induced premature senescence of MESCs

**Figure 1:**
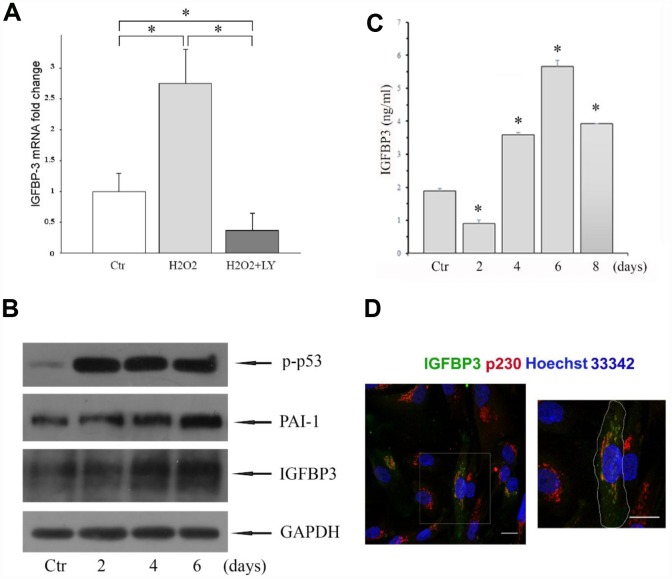
(**A**) Expression of *IGFBP3* gene was analyzed by qRT-PCR in MESCs treated with 200 μM H_2_O_2_ for 1 h (*H_2_O_2_*), or in LY-pretreated cells, stimulated with 200 μM H_2_O_2_ for 1 h, and then incubated with LY for 96 hours (*H_2_O_2_+LY*); *Ctr* – untreated cells. IGFBP3 mRNA was normalized to the reference gene SDHA, and results shown are relative to IGFBP3 expression in control cells. Data are presented as mean ± SD of duplicate determinations in two independent experiments. *p< 0.01. (**B**) Western blot analysis of the p-p53, PAI-1, and IGFBP3 protein expression in control (Ctr) and H_2_O_2_-treated cells. Cells were treated with 200 μM H_2_O_2_ for 1 h, and then re-cultured in fresh growth medium for the indicated time. GAPDH was used as a loading control. Representative results of three independent experiments are shown. (**C**) The IGFBP3 content in CM of control (Ctr) and H_2_O_2_-treated cells quantified by the ELISA. Analysis was performed at the indicated time after H_2_O_2_ treatment. Mean values ± SD of three independent experiments are shown, *p<0.01 (**D**) Young MESCs are heterogeneous by the level of endogenous IGFBP3 synthesis. Young cells were fixed and then stained for IGFBP3 antibodies (green), p230 (red) and Hoechst 33342 (blue). Images are presented as maximum intensity projections. Scale bars - 20 μm. Abbreviations: IGFBP3, insulin-like growth factor binding protein 3; MESCs, endometrial mesenchymal stem cells; LY (LY294002), a specific inhibitor of PI-3K; p-p53, phosphorylated p53; PAI-1, plasminogen activator inhibitor 1; GAPDH, glyceraldehyde 3-phosphate dehydrogenase; p230, a specific marker of trans-Golgi network; Hoechst 33342, Trihydrochloride, trihydrate, nuclear staining dye; qRT-PCR, quantitative real-time reverse transcription-polymerase chain reaction; ELISA, enzyme-linked immunosorbent assay; SD, standard deviation.

Primarily, by applying qRT-PCR and Western blot analysis we assessed the IGFBP3 expression levels in young (control) and senescent (H_2_O_2_-treated) MESCs. The expression levels of IGFBP3 mRNA (Figure 1A) and protein (Figure 1B) were higher in senescent cells than in young cells. The immunoblot analysis showed that both transcription factor p53 and plasminogen activator inhibitor 1 (PAI-1) were also upregulated in senescent cells (Figure 1B). The senescence development during 6 days was accompanied by gradually increase in the protein expression of IGFBP3 as well as PAI-1, suggesting that these endogenous proteins may mediate H_2_O_2_-induced senescence of MESCs.

To test the IGFBP3 content in CM from senescent MESCs, H_2_O_2_-treated cells were incubated in complete growth media for 2, 4, 6 or 8 days before CM harvesting at each time point. Then the IGFBP3 concentrations were determined by ELISA. As shown in Figure 1C, extracellular IGFBP3 levels increased in a time-dependent manner, peaking at 6 days. Of note, the experimental conditions such as a passage number, cell amount seeded, and a duration time of cell senescence before CM harvesting had a strong effect on extracellular IGFBP3 levels.

### Heterogeneity of intracellular IGFBP3 synthesis by young MESCs

The obtained results showed the presence IGFBP3 in CM of MESCs indicating that the cells synthesize IGFBP3 at some level. But is the capability to secrete IGFBP3 similar in all cells in population? To answer this question, we have conducted the immunofluorescent labeling of endogenous IGFBP3 in young MESCs. We have revealed that only about 11-14% of cells under the field of view show IGFBP3 antibody labelling indicating that the MESCs are heterogeneous by the level of endogenous IGFBP3 protein. Note that in IGFBP3-positive cells IGFBP3 is colocalized to a large extent with p230 (Figure 1D), a specific marker of trans-Golgi network that confirms IGFBP3 synthesis by these cells.

### Effect of the MAPK and PI-3K/Akt pathways inhibition on the IGFBP3 releasing from senescent MESCs

**Figure 2:**
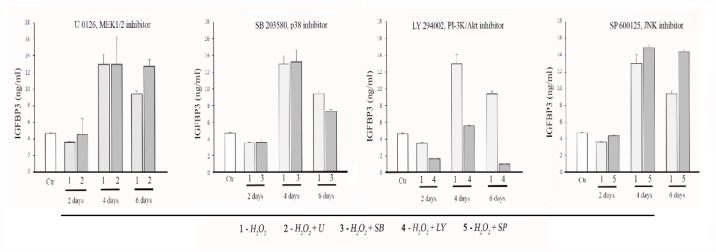
**Effects of the MAPK and PI3K pathway inhibitors on IGFBP3 releasing from MESCs in conditioned media (CM**). Cells were treated with 200 μM H_2_O_2_ for 1 h, or cells pretreated with one of specific inhibitors (10 μM U0126, 5 μM SB203580, 20 μM LY294002, 10 μM SP600125,) for 30 min were exposed to 200 μM H_2_O_2_ for 1 h, and then incubated with each of inhibitors for the indicated time; Ctr – untreated cells. The IGFBP3 concentration in CM was quantified by the ELISA. Mean values ± SD of three independent experiments are shown; p<0.01 for H_2_O_2_ (except 2 d) and each of inhibitors at the indicated time periods compared with Ctr (except U0126, 2 d and 4 d). Also, p<0.01 for LY at each time point, as well as for SP and SB at 6 day compared with H_2_O_2_ at the same time point.

Studying the molecular mechanism of H_2_O_2_-induced MESCs senescence, we have discovered that both MAP kinase and PI-3 kinase signal pathways are involved in regulation of MESCs premature senescence [52]. Therefore, we decided to examine an involvement of these pathways in IGFBP3 releasing from senescent cells. By utilizing the specific pharmacological inhibitors of ERK1/2, JNK and p38 MAP kinases, we did not observe any essential differences in the IGFBP3 secretion levels with exception of perceptible decrease in IGFBP3 under p38 MAPK inhibitor (Figure 2). In contrast, the PI-3K activity suppression by the specific inhibitor LY294002 (hereafter LY) abolished the IGFBP3 releasing from senescent MESCs. As seen in Figure 2, an inhibitory effect becomes most pronounced in 6 days after beginning the LY treatment. Also, the expression levels of IGFBP3 mRNA were strongly reduced in H_2_O_2_-stimulated cells after LY treatment (Figure 1A). The obtained results suggest that the PI-3K/Akt pathway function may positively regulate both IGFBP3 synthesis and IGFBP3 releasing from senescent MESCs.

### Inhibition of the PI-3K/Akt/mTOR pathway rescues MESCs from H_2_O_2_-induced premature senescence

Next, we addressed potential mechanisms underlying the LY effects observed. There is now increasing evidence that mTOR (mammalian target of rapamycin) pathway plays a crucial role in regulation of stem cell senescence program [53, 54]. The mTOR is an integral component of Ras/PI-3K/Akt/mTOR signaling cascade, and its function is regulated by upstream PI-3K/Akt kinases. In brief, Akt being a key mediator of PI-3K signal may activate mTOR complex 1 (mTORC1) which in turn activates downstream effector targets, notably eukaryotic translation initiation factor 4E-binding protein 1 (4E-BP1) and S6 kinase 1 (S6K1). Activation of mTOR is known to be required for acquiring senescent phenotype. Since PI-3K is an upstream activator of mTOR, it was expected that LY would decelerate senescence or at least prevent emerging senescence-related phenotype of MESCs.

**Figure 3:**
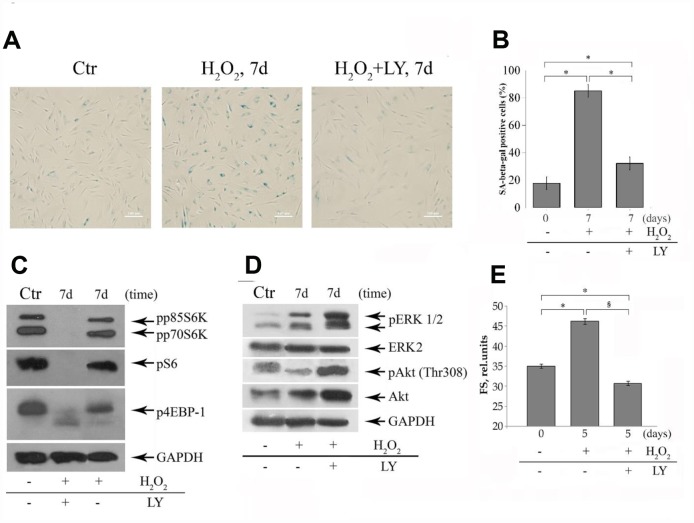
**The LY treatments cause modulation of both senescent phenotype and the phosphorylation status of mTOR targets and ERK1/2 MAPK in senescent MESCs.** (**A**) SA-β-Gal staining 7 days after 1h-H_2_O_2_-treatment (middle) or after (H_2_O_2_+LY) treatment as described in the legend of Figure 2 (right). Ctr – control cells. Senescent cells were detected with SA-β-Gal staining kit. Ob: 10x; scale bars: 140 μm. (**B**) Quantitative assay of SA-β-Gal positive cells*.* Data are presented as mean ± SD*,* p < 0.001. (**C**, **D**) The expression levels of mTORC1 targets (p70S6K, S6, 4EBP-1), Akt, and ERK1/2 were revealed by immunoblot analysis 7 days after H_2_O_2_ or (H_2_O_2_+LY) treatment as indicated in (A). GAPDH was used as a loading control. Ctr – untreated cells. Representative results of the three experiments are shown in the figures. (E) Forward scatter (FS), reflecting the average cell size was measured by light-scattering cytometry 5 days after H_2_O_2_ or (H_2_O_2_+LY) treatment as indicated in (A). The results are presented as mean ± SD of three independent experiments, p<0.01 compared to control () and H_2_O_2_-treated cells (§). Abbreviations: pp85S6K, phospho-p85 S6 kinase (Thr412); pp70S6K, phospho-p70 S6 kinase (Thr389); pS6, phospho-S6 ribosomal protein (Ser240/244); p4EBP-1, phospho-4EBP-1 (Thr37/46); pAkt, phospho-Akt (Thr308); pERK1/2, phospho-ERK1/2 (Thr202/Tyr204).

To examine whether Akt is implicated in controlling functional activity of mTOR pathway during H_2_O_2_-induced senescence of MESCs, the LY effects on phosphorylation status of mTORC1 targets were tested. The permanent LY post-treatment of H_2_O_2_-stimulated cells abrogated phosphorylation of S6K, S6 and 4E-BP1 compared to control and H_2_O_2_-treated (senescent) cells (Figure 3C), while increasing phosphorylation levels of Akt and ERK1/2 MAP kinases (Figure 3D). Increased Akt phosphorylation correlated with enhanced Akt protein expression. As for ERK, similar effect of rapamycin, another specific mTORC1 inhibitor, increasing ERK activity through S6K/PI3K/Ras-dependent feedback loop, was revealed in both normal and cancer cells [55]. These findings are in line with our results, demonstrating a strong ERK1/2 phosphorylation increase in rapamycin-treated MESCs after senescence induction (data not shown). It cannot exclude that LY-induced mTORC1 inhibition also might mediate the ERK activity augmentation with senescence.

Further, to verify whether the PI-3K/Akt/mTOR inhibition affects modulation of senescent phenotype, hallmarks typical of senescent cells such as enlarged and flattened morphology, increased cell size and senescence-associated β-galactosidase (SA-β-Gal) activity were evaluated. As expected, H_2_O_2_-stimulated (senescent) cells showed the characteristic senescent phenotypes (Figure 3A) that distinguished themselves from the control cells, while the LY post-treatment significantly decreased a number of SA-β-Gal stained cells (Figure 3B) and altered cell morphology (Figure 3A) as well as prevented cell hypertrophy (Figure 3E). Taken together, these findings evidence that LY indeed prevents emerging the senescence phenotype through PI-3K/Akt/mTOR suppression in H_2_O_2_-stimulated cells. The observed LY effect seems to ensure a delay and ultimately prevention of premature senescence of stressed cells, thus hindering IGFBP3 upregulation.

### IGFBP3 is an important factor of senescent CM for premature senescence induction in young MESCs

**Figure 4:**
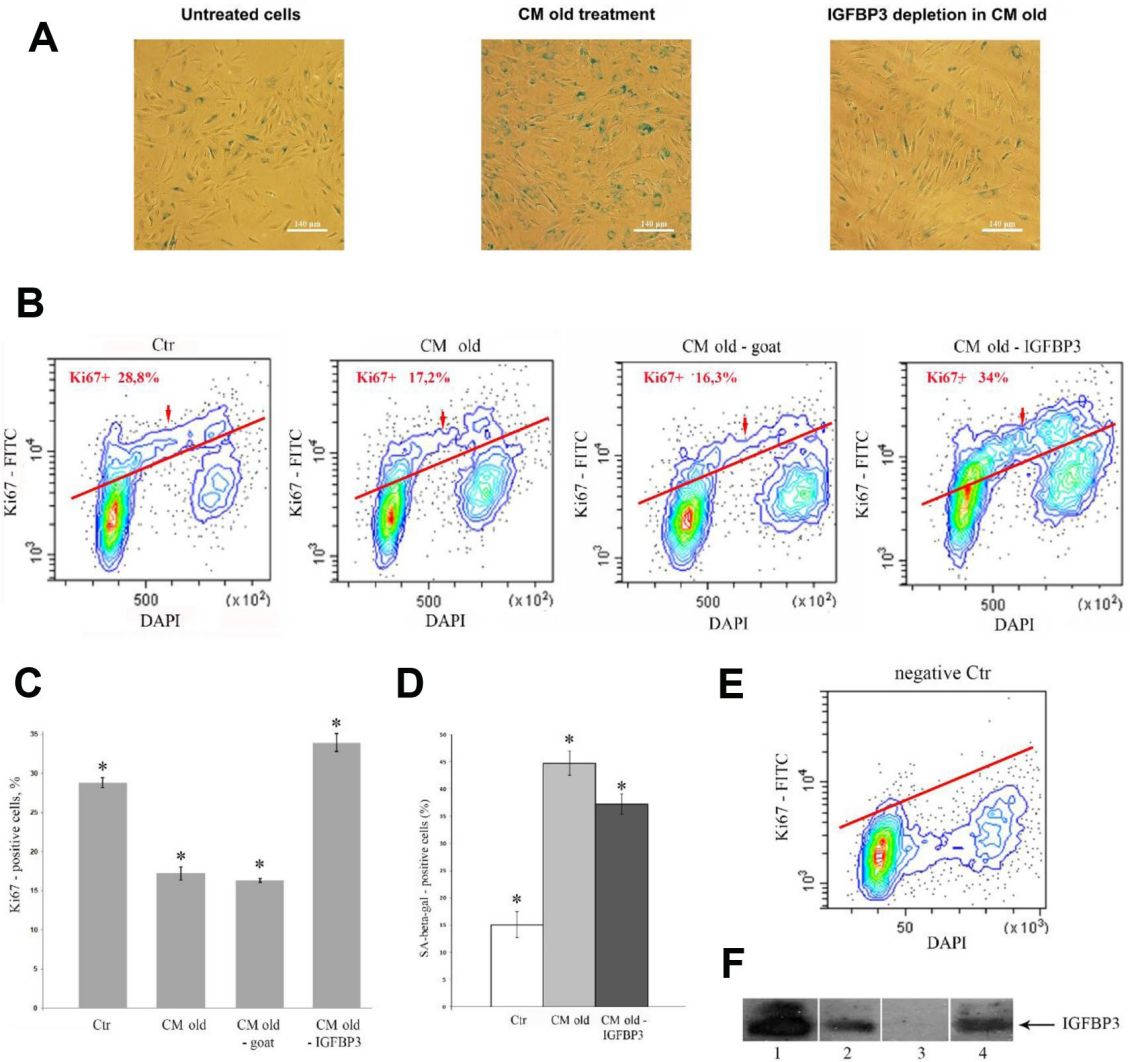
**Effects of the IGFBP3-depleted CM old on young MESCs.** (**A**, **D**) IGFBP3 immunodepletion decreases a population of SA-β-Gal positive cells. (**A**) SA-β-Gal staining 9 days after CM old treatment (middle) or after the IGFBP3-depleted CM old treatment (right). Representative microphotographs are shown. Ob: 10x; scale bars: 140 μm. (**D**) Quantitative assay of SA-β-Gal positive cells*.* Data are presented as mean ± SD*,* *p < 0.05. (**B**, **C**, **E**) The IGFBP3 immunodepletion increases the proliferation of young MESCs. (**B**) Ki67 staining. Cells were treated with indicated CMs for 9 days, stained with FITC-Ki67 conjugate and DAPI, and analyzed by FACS. (**E**) The negative control (negative Ctr) - FITC Mouse IgG1 staining. The representative FACS contour-plots of stained MESCs are shown; red arrows indicate S-phase cells. (**C**) Quantitative assessment of Ki67 positive cells. Ctr – untreated cells. The means ± SD of three independent experiments are presented, *p < 0.05. (**F**) Testing of IGFBP3 content in CMs by immunoblotting. 1 – recombinant IGFBP3, positive control; 2 – CM old after immunoprecipitation with normal goat IgG control antibodies, negative control; 3 – CM old after immunoprecipitation with specific IGFBP3 antibodies (IGFBP3-depleted CM old); 4 – CM from H_2_O_2_-treated (senescent) cells (CM old).

To determine the role of extracellular IGFBP3 for triggering senescence in young cells, it was eliminated from senescent CM by means of immunoprecipitation with specific anti-IGFBP3 antibody. The IGFBP3 immunodepletion completeness was confirmed by CM immunoblot analysis (Figure 4F). To make sure of the IGFBP3 depletion specificity, the senescent CM was incubated with the control normal goat IgG in parallel. In this case, we observed only minor, if any, decrease in the IGFBP3 content in CM, as compared to CM collected from senescent (H_2_O_2_-treated) cells. After prolonged incubation of young MESCs with IGFBP3-immunodepleted CM, the main senescence hallmarks such as SA-β-Gal activity (Figure 4A, 4D) and the cell proliferative potential (Figure 4B, 4C, 4E) were assayed.

As shown in Figure 4D, a proportion of SA-β-Gal-positive cells was lower after incubation of young cells in IGFBP3-depleted CM compared to senescent CM. A proliferative status of cells was examined by staining with antibodies against proliferation marker Ki67. Unlike the senescent CM, the incubation of young cells with IGFBP3-depleted CM caused noticeable increase in the number of Ki67-positive cells in the cell culture (Figure 4C). Of note, the effects of both senescent CM and goat IgG-depleted CM on proliferative status of young MESCs were alike. Thus, the IGFBP3 immunodepletion reduced the pro-senescent activity of senescent CM and promoted cell growth.

**Figure 5:**
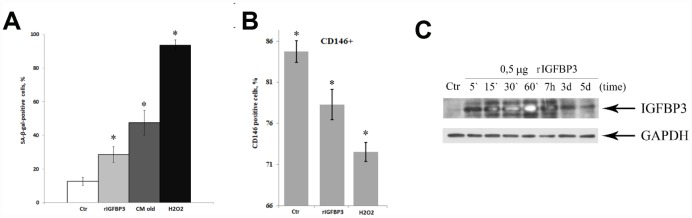
**MESCs acquire the senescent phenotype in response to recombinant IGFBP3.** (**A**) Quantitative assay of SA-β-Gal positive cells after treatment with 0.5 μg rIGFBP3 or CM old for 9 days, or 200 μM H_2_O_2_ for 1 h. Senescent cells were detected with SA-β-Gal staining kit. The results are presented as mean ± SD of three independent experiments, *p<0.01. (**B**) Analysis of the MCAM/CD146 expression levels. Quantitative assay of CD146 positive cells after treatment with 0.5 μg rIGFBP3 for 9 days or 200 μM H_2_O_2_ for 1 h. Mean values ± SD of three independent experiments are shown, *p<0.05. (**C**) rIGFBP3 internalization dynamics. Cells were stimulated with 0.5 μg rIGFBP3 for the indicated time, and then IGFBP3 expression levels were analyzed by immunoblotting in each time point. GAPDH was used as a loading control. Representative results of three independent experiments are shown. Ctr – untreated cells. Abbreviation: MCAM/CD146, the melanoma cell adhesion molecule.

The involvement of IGFBP3 in senescence promotion of MESCs was further confirmed by incubating young MESCs with human recombinant IGFBP3 protein (rIGFBP3). A long-term treatment with 500 ng/ml rIGFBP3 increased the percentage of SA-β-Gal-positive cells with respect to control cells (Figure 5A). Insignificant increase in SA-β-Gal staining, if any, was observed at lower protein concentration used (100 ng/ml) (data not shown). As expected, SA-β-Gal activity of cells incubated with the senescent CM or exposed to H_2_O_2_ was much higher. In this set of experiments, H_2_O_2_ being a strong inducer of MESCs premature senescence served as a positive control. Interestingly, rIGFBP3-stimulated cells in 3 days after beginning treatment showed 1.5-fold higher autofluorescence than control cells (data not shown). Earlier we demonstrated that stress-induced senescence of MESCs is accompanied by an increase in autofluorescence which is a consequence of the accumulation of lipofuscin granules [56, 57]. Since the accumulation of lipofuscin is one of the senescence markers, the cell autofluorescence enhancement in response to exogenous rIGFBP3 may indicate the senescence phenotype.

In addition, we analyzed the rIGFBP3-induced expression alteration of the melanoma cell adhesion molecule (MCAM/CD146), one of the MSC-surface markers related to the cellular senescence. As indicated in Figure 5B, a proportion of CD146-positive cells markedly decreased compared with control cells following prolonged rIGFBP3 treatment while in H_2_O_2_-treated cells the observed lowering the CD146 expression was more pronounced. The presented results suggest that MESCs acquire the senescence phenotype in response to exogenous rIGFBP3. As expected, H_2_O_2_ was more effective at inducing senescence phenotype.

Together, the data obtained assume that, among SASP factors contained in senescent CM, the IGFBP3 appears to be an important factor involved in senescence induction of young MESCs due to its paracrine action.

### Dynamics of rIGFBP3 internalization in young MESCs

The observed effects of both IGFBP3-depleted CM and rIGFBP3 on young MESCs imply the extracellular IGFBP3 penetration into cells that is required to its function. To test this hypothesis, first dynamics of rIGFBP3 internalization in young cells was explored by immunoblotting. As shown in Figure 5C, cell treatment with 0,5 μg rIGFBP3 for 1 h resulted in gradual enhancing the intracellular IGFBP3 levels in a time-dependent manner. Of note is that dynamics pattern was independent of rIGFBP3 concentration tested in the range from 0,1 μg to 0,5 μg.

Having established the fact of intracellular IGFBP3 increase in MESCs during 1 h rIGFBP3 treatment, we next decided to examine whether exogenous IGFBP3 undergoes internalization in these cells by endocytic pathway. Using immunofluorescent staining, we have studied the dynamics of exogenous rIGFBP3 internalization in young cells and the colocalization of this protein with the marker of canonical early endosomes EEA1. EEA1-positive endosomes are well known to be the first sorting hub in the endocytic pathway, where cargoes are sorted to the two main pathways: degradative pathway ending in the lysosomes and the recycling pathway that deliver cargoes back to the plasma membrane [58].

**Figure 6:**
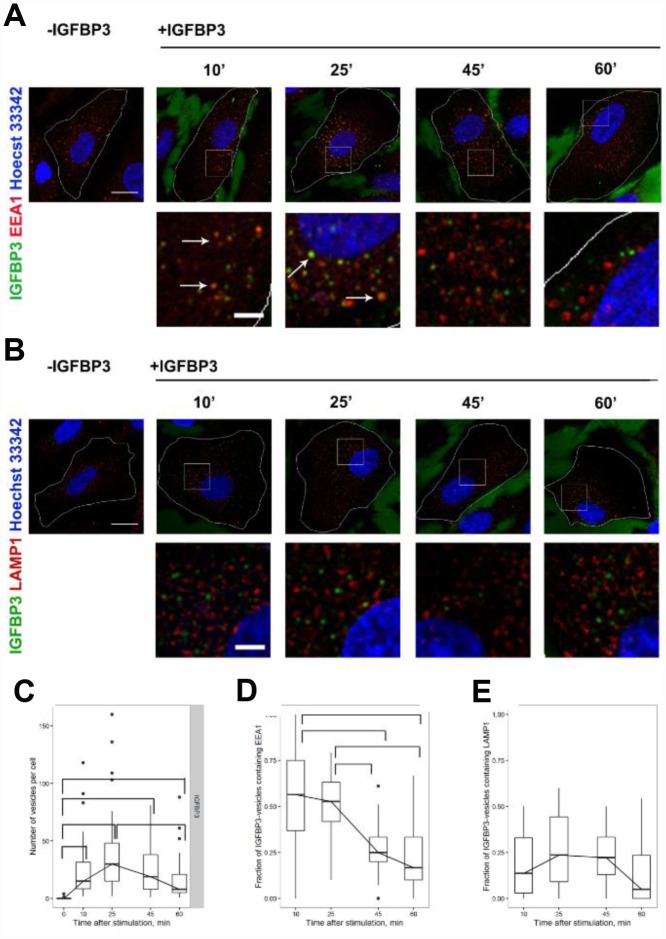
**The analysis of exogenous rIGFBP3 colocalization with EEA1-positive early endosomes and LAMP1-positive late endosome/lysosomes.** Exogenous rIGFBP3 was added to young MESCs for 10 min as indicated in Materials and Methods. Then the IGFBP3-treated cells (+IGFBP3) as well as control cells (-IGFBP3) were fixed and stained for IGFBP3 antibodies (green), EEA1 (**A**, red) or LAMP1 (**B**, red) and Hoechst 33342 (blue). White arrows indicate structures with the colocalization of IGFBP3 and endosome marker signals. Images are presented as the maximal intensity projections of three consecutive optical slices. Scale bars: 20 μm (upper panels) and 5 μm (lower panels). On the base of immunofluorescent images, the number of IGFBP3 vesicles (**C**) as well as the fraction of IGFBP3 vesicles containing EEA1 (**D**) or LAMP1 (**E**) were calculated. Data are presented as boxplots with median, interquartile range, minimum and maximum*. Parentheses* indicate statistical differences (p<0.05).

rIGFBP3 (1μg/ml) was added to cells for 10 min at 37°C and then washed out to achieve simultaneous internalization of cargo that allows to examine consistently different stages of rIGFBP3 intracellular transport. 10 min after rIGFBP3 addition about 28+/-6 rIGFBP3-positive vesicles were found in cells (Figure 6C). The maximum number of rIGFBP3-vesicles (42+/-8) was detected at 25 min with subsequent decreasing at 45-60 min to 17+/-4. rIGFBP3-vesicles were distributed throughout the cell at different time points with no obvious tendency to peripheral or juxtanuclear localization (Figure 6A, 6B). It must be noticed that, besides the intracellular IGFBP3, we have found a considerable background IGFBP3 antibody labelling of intercellular space (Figure 6A, 6B). As without exogenous IGFBP3 addition (control) no IGFBP3 labelling was detected, we conclude that this background is due to exogenous IGFBP3 protein but not non-specific antibody binding. According to literature data, IGFBP3 interacts with multiple matrix proteins including heparin, fibronectin, fibrin, cadherin, and collagen [32, 59] that can explain the existence of such IGFBP3-dependent background. Of note, though using the lower IGFBP3 concentration (0,1-0,5 μg) led to decreasing the background, the endosomes’ brightness was also reduced that impeded their detection.

Concerning the colocalization of intracellular rIGFBP3 with early endosome marker EEA1, the maximal fractions of IGFBP3-vesicles containing EEA1 were detected at early time points (10-25 min) with subsequent decreasing at 45, 60 min (Figure 6D). According to these data, it may be supposed that the cargo (rIGFBP3) at 45-60 min is transported from early to late endosomes and then to lysosomes for degradation. To check this hypothesis, we additionally analyzed the colocalization of rIGFBP3 with the marker of late endosomes and lysosomes LAMP1 (Figure 6B, 6E). LAMP1-postive structures was found to be distributed throughout the cell with the predominantly juxtanuclear localization (Figure 6B). There was a little colocalization of rIGFBP3 with LAMP1 (about 20%) at all time points investigated with no changes in colocalization dynamics over time (Figure 6E). Together, these results indicate that rIGFBP3 undergoes rather recycling than degradation in LAMP-positive endosomes.

## DISCUSSION

Earlier we have shown that SASP factors released from MESCs in response to oxidative stress trigger the premature senescence program within young cells [49]. By using a secretome proteomics approach, we have analyzed a composition of senescent MESCs secretome and elucidated up-regulation of both IGFBP3 and PAI-1 which displayed 2.28- and 2.26-fold increased levels in CM of senescent MESCs [57]. These findings prompted us to investigate a possible role for IGFBP3 as a mediator of the paracrine senescence of young MESCs. Several previous studies showed that IGFBP3 expression and secretion increase upon replicative senescence of various primary cell types [21, 60] or upon different senescence-inducing stresses such as hypoxia, etoposide, UV, ionizing radiation, and H_2_O_2_ [23]. Gradually increased accumulation of IGFBP3 in CM of senescent MESCs in response to oxidative stress is fully consistent with these observations. The expression levels of IGFBP3 mRNA and protein, as evident by both specific immunoblots and qRT-PCR, were also significantly enhanced in MESCs with senescence.

By applying inhibitory analysis to study the regulation mechanism of IGFBP3 secretion by senescent MESCs, the important results were obtained. According to our data, the functional activity of PI-3K/Akt/mTOR, but not MAPK pathways, is required for IGFBP3 secretion. In H_2_O_2_-stimulated MESCs, a permanent inhibition of PI-3K suppressed the mTOR pathway activity that is crucial to emerging senescence phenotype. Similar LY effects on mTOR functional state were described for different types of human cancer and normal cells including MSC [53, 54]. As well, the main features of senescent phenotypes such as cell hypertrophy and increased SA-β-Gal activity were strongly diminished in (H_2_O_2_+LY)-treated MESCs, unlike H_2_O_2_-treated. Inhibition of Akt/mTOR by a long-term LY treatment was shown to reduce ROS production in MSC aged in vitro [54]. There is perhaps a crosstalk between mTOR and ROS pathways suggesting reciprocal regulation of these pathways by each other. In that case, mTOR inhibition might decelerate the oxidative DNA damage accumulation, thus allowing to delay or prevent the appearance of senescence phenotypes. We speculate that a long-term LY treatment of MESCs exposed to oxidative stress rescues cells from premature senescence thereby hindering IGFBP3 secretion by a so far unknown mechanism. We believe that there should be a correlation between senescence progress and IGFBP3 releasing from senescent MESCs into CM.

Accumulating evidence indicate that IGFBP3 implicated in extracellular cascade of secreted proteins plays an essential role in the regulation of cellular senescence. It is noteworthy that extracellular IGFBP3 levels increased upon various senescence inducing stimuli, and IGFBP3 induced senescence in different cell types [21, 23, 24, 61]. In keeping with these observations, the paracrine/autocrine activity of IGFBP3 is assumed to underlie its senescence-inducing effects. To our knowledge, there are no reports of exogenous IGFBP3 in association with premature senescence induction in MESCs cultures. Here, we have demonstrated for the first time a role for IGFBP3 as a secreted mediator of MESCs premature senescence. According to our data, IGFBP3 is involved in the senescent effect induction, modulating the functions of young MESCs. So, IGFBP3 elimination from senescent CM attenuated its pro-senescent effect on young MESCs, promoting cell growth and reducing SA-β-Gal activity. In contrast, prolonged treatment of young cells with rIGFBP3 increased SA-β-Gal activity, and at the same time decreased the expression of CD146/MCAM which is considered a specific MSC marker [62]. It should be noted that long-term observations were required to estimate the MESCs responses to the treatment with depleted CM. Generally, our findings are consistent with previous studies, demonstrating the role of exogenous IGFBP3 as a mediator doxorubicin-induced senescence of breast cancer cells [23], as well as replicative senescence of umbilical vein endothelial cells [21, 61]. As reported recently, the secretome analysis of the bone marrow and adipose MSC with various stress-induced phenotypes permitted to identify the common p53/PAI-1/IGFBP3 signaling pathway mediating the senescent signal transmission to surrounding cells [24]. Consistent with our results, each of the above proteins was upregulated in H_2_O_2_-stimulated MESCs with senescence, therefore it is likely that the same pathway could be responsible for paracrine senescence induction within young MESCs. This hypothesis needs future testing.

The results obtained in this work clearly demonstrate that exogenous IGFBP3 is effectively internalized into young MESCs and persists for quite long period (about 30 min) within early endosomes. rIGFBP3 doesn’t seem to enter the degradative pathway as there is no tendency to increase rIGFBP3 colocalization with LAMP1 over time. Alternatively, rIGFBP3 could apparently undergo recycling in MESCs as the number of rIGFBP3-bearing vesicles decreases by 60 min. As IGFBP3 bears NLS, it is assumed by some authors that IGFBP3 is able to enter the cells via the endocytic pathway, escape from the endosomes to the cytosol and then enter the nucleus [6]. Based on the immunofluorescent images, we cannot definitely assert if there is any exogenous IGFBP3 translocation into the nucleus of MESCs. By now, the mechanism of protein cargo escape from endosomes remains to be unknown. Generally, the cargoes itself do not need to be translocated to the nucleus to perform its function as endosomes are well known to be the platforms for signal cascades assembly and amplification [63]. The question of what cellular compartments use IGFBP3 to translate its «message» remains to be the subject of future debates.

In conclusion, progress observed in regenerative medicine in recent years is connected with development of the new MSC-based strategies, including 3D bioprinting or self-assembly, recapitulating organs and tissues via scaffold making [64, 65]. However, the comprehensive study of mechanism underlying a direct administration of undifferentiated MSC which may migrate to the lesion areas with following differentiation and substitution of damaged cells remains the issue of the day. At present, it is recognized that along with multipotent differentiation potential of MSC their strong paracrine capacity may play a decisive role in tissue repair. Although MSC offer great promise in the treatment of degenerative diseases and inflammatory disorders [38–42, 47, 48], there are more problems to be solved before their widespread clinical use. In particular, the precise paracrine mechanism of MSC action is far from being completely understood, and remains the active debate subject [38–42]. In addition, a phenomenon of oxidative stress-induced senescence that significantly modulates the cell secretory activity promoting the paracrine senescence of surrounding cells is necessary to consider for beneficial effects of MSC-based therapy.

## MATERIALS AND METHODS

### Cell cultures

Human endometrial mesenchymal stem cells (MESCs) were derived from a desquamated endometrium contained in menstrual blood of healthy donors [44]. All donors of menstrual blood signed an informed consent for voluntary participation. The samples were processed in accordance with the ethical committee of the Institute of Cytology of Russian Academy of Sciences and the principles of the Declaration of Helsinki. MESCs possess properties typical for the mesenchymal stem cell cultures: cells express CD13, CD29, CD44, CD73, CD90, and CD105 surface markers, and are negative for the hematopoietic markers CD45 and CD34 [44, 45]. Multipotency of isolated MESCs is confirmed by their ability to differentiate into other mesodermal cell types, such as osteocytes and adipocytes [44, 45]. Cells were cultured according to a standard protocol in DMEM/F12 medium (Gibco BRL, USA) supplemented with 10% FBS (HyClone, USA), 1% penicillin-streptomycin (Gibco BRL, Gaithersburg, MD, USA) and 1% GlutaMax (Gibco BRL, USA). Before experiments, cells were harvested by trypsinization and plated at a density of 15x10^3^ cells/cm^2^. For microscopy experiments, cells were grown on glass coverslips at seeding density 6-7x10^4^ cells/cm^2^. Cells from the 5-10^th^ passages were used in all experiments.

### Cell treatments

H_2_O_2_-treated cells and control (H_2_O_2_-untreated) cells were referred to as senescent (or old) and young MESCs, respectively. To induce the premature senescence, the cells were treated with H_2_O_2_ (Sigma, St. Louis, MO, USA) as reported previously [50]. Briefly, the cells exposed to 200 μM H_2_O_2_ for 1 h were washed twice with serum-free medium to remove H_2_O_2_, and re-cultured in fresh complete growth medium for 7 days [49, 51]. In indicated cases, before H_2_O_2_-stimulation cells were pretreated with one of kinase inhibitors (5 μM SB203580, 10 μM SP600125, 10 μM U0126, 20 μM LY294002) for 30 min, and then were incubated in the presence of these inhibitors during various time as specified in individual experiments. Recombinant human IGFBP3 (rIGFBP3, R&D Systems, USA) was added to young MESCs cultures at a final concentration of 0.5-1 μg/ml, and was present within incubation media during all period of observation with the exception of IGFBP3 internalization assay.

### Preparation of conditioned media and measurement of IGFBP3 concentration

The preparation of conditioned media (CM) was performed as reported previously [49]. Finally, the senescent cells were washed twice with PBS and re-cultured in serum-free medium for 24 h. The CM collected from senescent cells is referred to as CM-old. The IGFBP3 concentration in CMold was measured with Quantikine ELISA Human IGFBP3 kit (R&D Systems, USA). To prepare IGFBP3-depleted CMold, IGFBP3 was immunodepleted in CMold by incubating CM and cells with a polyclonal anti-human IGFBP3 (R&D Systems, USA) at a final concentration of 16 μg/ml for 2 h at 4°C. Likewise, the normal goat IgG (R&D Systems, USA) were used for control experiments. IGFBP3-depleted CMold is referred to as CMold-IGFBP3 whereas normal goat IgG depleted CMold – as CMold-goat. After the immunoprecipitation procedure fulfillment, both CM were used at 50% in complete growth medium for in vitro cultivation of young MESCs during various time as specified in individual experiments.

### SA-β-Gal activity assay

Cells expressing senescent-associated β-galactosidase (SA-β-Gal) were detected with senescence β-galactosidase staining kit (Cell Signaling Technology) according to manufacturer’s instructions and quantified microscopically by counting X-gal-positive cells among not less 500 cells in random fields of view [66].

### FACS analysis of cell size (1), Ki67/DAPI staining (2), CD146 staining (3)

**(1)** Cells were detected by size and granularity using FSC/SSC, and cell debris was gated out. The cell size was evaluated by cytometric light scattering of PI-negative stained cells [67]. To discriminate the live and dead cells, two-parameter histogram (DotPlot or Cytogram) was used (FL4LOG vs. FSLOG). **(2)** The samples were prepared using Nuclear Factor Fixation and Permeabilization Buffer Set (BioLegend, USA). Briefly, after fixation and permeabilization cells were stained with FITC-Ki67 conjugate (Dako) (10 μl/10^6^ cells) and DAPI (1 μg/ml). FITC Mouse IgG1 (BD Pharmingen) served as a negative control [68, 69]. **(3)** After trypsinization and washing with PBS, MESCs were concentrated to 1x10^6^ cells per ml in FACS buffer, and then stained with CD146 antibody (Beckman Coulter, USA) conjugated with phycoerythrin in accordance with manufacturer’s recommendations at least for 40 min at +4°C in the dark. Phycoerythrin conjugated Mouse IgG1 used as isotype control. The percentage of expressed cell surface antigens was calculated for 10, 000 gated-cell events [62, 70, 71]. In all indicated cases, flow cytometry was performed using the CytoFLEX (Backman Coulter, CA, USA), and the obtained data were analyzed using CytExpert software version 2.0.

### Western blotting

Preparation of total cell lysates and Western blot analysis were performed as described previously [48]. Protein content was detected using Bradford method. SDS-PAGE electrophoresis, transfer, immunostaining, and ECL detection were carried out according to standard protocols of Bio-Rad Laboratories and antibody manufacturers. Primary antibodies against the following proteins were utilized: IGFBP3 (B-5, 1:500, from Santa Cruz Biotech.), phospho-p70S6 kinase (Thr389, 1:1000), phospho-S6 (Ser240/244, 1:1000), phospho-4E-BP1 (Thr37/46, 1:1000), phospho-ERK1/2 (Thr202/Tyr204, 1:2000), ERK2 (1:3000), phospho-Akt (Thr308, 1:700), Akt (1:1000), and GAPDH (clone 14C10, 1:4000) – all from Cell Signaling Technology. Secondary antibodies for immunoblotting – GAR-HRP (1:10000) and GAM-HRP (1:10000) were also from Cell Signaling Technology.

### qRT-PCR assays

To analyze gene expression, total RNA was isolated by RNeasy Mini Kit (Qiagen) from both untreated (control) and treated with H_2_O_2_ or (H_2_O_2_+LY) cell cultures. Two biological replicates for each experiment were performed. First-strand cDNA was synthesized from 1.5 μg of total RNA by using 1 μg of random hexamers, 100 units of MMLV reverse transcriptase, 0.5 mM dNTPs, and 1× MMLV buffer (Silex, Russia) in a total volume of 20 μl at 37°C for 1 h. In negative control experiments MMLV reverse transcriptase was omitted. The PCR primers for IGFBP3 were designed using the GeneRunner v 5.0.59 software. To avoid false positive results due to genomic contamination of the samples, the primers spanned an intron at the genomic level. The primer sequences used for IGFBP3: 5`-TCACCTGA AGTTCCTCAATGT-3’ (forward) and 5`-ACTTATCCACACACCAGCAGA-3’ (reverse), expected amplicon length is 137 bp, for reference gene SDHA: 5`-CCACTCGCTATTGCACACC-3’ (forward) and 5`-CACTCCCCGTTCTCCATCA-3’ (reverse), expected amplicon length is 102 bp Primers were synthesized by the Syntol (Russia).

qRT-PCR was performed using the CFX96 Real-Time PCR Detection System (Bio-Rad Laboratories, Hercules, CA) [72] in duplicate for each transcript in 10 μl mixtures, containing 2 μl diluted (1:4) cDNA, 0.5 μM of each primer, 200 μM dNTPs, 2 mM MgCl2, 1×Hot-Taq SYBR Green I polymerase buffer (Syntol, Russia) and 1 unit Hot-Taq polymerase (Syntol, Russia). The PCR cycling conditions were 8 min at 94°C; 40 cycles of 40 s at 94°C, 60 s at 60°C; data collection was at the end of each 60°C phase. To estimate PCR efficiencies, standard curves were generated using four-fold serial dilutions of a cDNA, prepared from control cells. Estimated efficiency was 96% for IGFBP3, R^2^ 0.99 and 94% for SDHA, R^2^ 0.99. Quantification of IGFBP3 mRNA fold change was assessed using the 2^-(ΔΔCt)^ method [73]. Significance of fold change estimates was determined with the independent-sample t-tests.

### IGFBP3 internalization assay

To conduct immunofluorescence staining, cells were seeded onto 10×10 coverslips. The seeding density was 6-7*10^4^ cells/cm^2^ to achieve about 90% monolayer at the day of experiment. 24 h later cells were washed twice with warm (37°C) DMEM/F12 medium and then endocytosis was stimulated by addition of 1μg/ml IGFBP3 (R&D Systems, USA) in DMEM/F12 medium (Gibco, USA) at 37°C for the time indicated.

### Immunofluorescence staining

In the indicated time points after IGFBP3 addition, cells were washed with warm PBS to remove unbound IGFBP3. For the immunofluorescence staining, cells were fixed with 4% formalin in PBS and permeabilized with 0.5% Triton X-100 or, in case of LAMP1-staining, with 0.05% Brij-56 for 15 min at room temperature (RT). Then cells were incubated with 1% BSA for 30 min at RT and treated with following primary antibodies: goat polyclonal anti-IGFBP3, dilution 1:50 (R&D Systems, USA), mouse monoclonal anti-EEA1, dilution 1:200 (Transduction Lab, USA), mouse monoclonal anti-LAMP1, dilution 1:50 (Abcam, UK), and mouse monoclonal anti-p230, dilution 1:50. (Transduction Lab, USA) either overnight at 4°C (for IGFBP3, LAMP1 and p230) or for 1 h at RT (for EEA1). After washing with PBS, cells were incubated with secondary antibodies DAG Alexa Fluor 488 (Molecular Probes, USA), dilution 1:50 and RAM Alexa Fluor 568 (Molecular Probes, USA), dilution 1:100. Antibodies were diluted in PBS with 1% BSA. Simultaneously with the solution of secondary antibodies, 5 μg/ml Hoechst 33342 solution (Thermo Fisher Scientific, USA) was added to cells. Finally, cells were washed with PBS and mounted in 0.2 M DABCO (Sigma, USA) glycerol-containing media.

### Image acquisition

Images were obtained using Leica TCS SP5 confocal microscope (Leica Microsystems, Germany) with 40X oil objective (NA=1.25). Series of optical sections (z-stacks) were collected with 0.5 μm step. The image size was 1024 X 1024 pixels. DAG Alexa Fluor 488 fluorescence was excited with argon laser (488 nm, detection range 500—560 nm). RAM-Alexa Fluor 568 was excited with He-Ne laser (543 nm, detection range 580—650 nm). Hoechst 33342 fluorescence was excited with diode laser (405 nm, detection range 420—470 nm). Fluorescence at every wavelength was scanned separately using Leica Confocal Software. 3-5 fields were registered in XYZ projections. Presented images are typical for most cells in the experiment. Images presenting LAMP1 staining were processed using pre-processing method prior the segmentation as described in Image processing and analysis section [74].

### Image processing and analysis

The number of vesicles and its parameters were evaluated using ImageJ 1.40 g (National Institute of Health, USA). 8-bit maximal intensity projections of a z-stack series were taken into analysis. Spatial calibration of images was conducted using «Set Scale» function. Segmentation was performed as described previously [74] with some modifications. Median filter with the radius value 6 for green channel and 10 for red channel (values were matched experimentally) was used and obtained image was subtracted from the initial one. As the result the difference between the vesicle edge pixel intensity and adjoining background pixel intensity was larger at the obtained image compared with the initial one. This allowed avoiding the artificial enlargement of vesicle size upon image binarization. Then obtained image was binarized using the threshold values which provided the maximal correspondence between the original and binarized image. Regions of Interest (ROI) were selected with the use of «Analyze Particles» function, so that ROI corresponded to vesicles, whose area were larger than 20000 nm2.

Obtained selections were restored on the initial image and used for measuring vesicles parameters. Such parameters as the number of vesicles per cell and the mean integral density of each vesicle were estimated on the base of maximal intensity projections of optical sections. To evaluate the object-based colocalization the same segmentation procedure was used on the maximal intensity projections of three consecutive optical slices. These slices represented base and middle planes of the cell, where the most of IGFBP3-containing vesicles are localized. The number of overlapping objects was estimated by superposition of ROIs from one channel to the binarized image of another channel. The object-based colocalization values are presented as the percentage of the number of overlapping objects from the total number of objects.

### Statistical analysis

All data are presented as the mean values of at least three independent experiments with standard deviations if other is not indicated. Statistical significance was evaluated using Student’s t-test or χ^2^ criterion, and p-values < 0.05 were considered significant. The immunofluorescence results were analyzed with R software (R Core Team, 2015) and statistical significance was validated using the Mann-Whitney U-test for independent samples.
